# Electropolymerised Polypyrroles as Active Layers for Molecularly Imprinted Sensors: Fabrication and Applications

**DOI:** 10.3390/ma14061369

**Published:** 2021-03-11

**Authors:** Karolina Glosz, Agnieszka Stolarczyk, Tomasz Jarosz

**Affiliations:** Department of Physical Chemistry and Technology of Polymers, Silesian University of Technology, 44-100 Gliwice, Poland; karolina.glosz@polsl.pl (K.G.); agnieszka.stolarczyk@polsl.pl (A.S.)

**Keywords:** polypyrrole, molecularly imprinted, sensor, electropolymerization

## Abstract

Conjugated polymers are widely used in the development of sensors, but even though they are sensitive and robust, they typically show limited selectivity, being cross-sensitive to many substances. In turn, molecular imprinting is a method involving modification of the microstructure of the surface to incorporate cavities, whose shape matches that of the “template”—the analyte to be detected, resulting in high selectivity. The primary goal of this review is to report on and briefly explain the most relevant recent developments related to sensors utilising molecularly imprinted polypyrrole layers and their applications, particularly regarding the detection of bioactive substances. The key approaches to depositing such layers and the most relevant types of analytes are highlighted, and the various trends in the development of this type of sensors are explored.

## 1. Introduction

Molecular imprinting is a group of processing methods that produce matrices that are riddled with cavities, whose shape is complementary to that of a “template” molecule. The interior of these cavities is often equipped with functional groups compatible with and capable of bonding with the functional groups of the template. The focus of molecular imprinting is primarily on polymer matrices, even though other types of matrices, such as TiO_2_ have also been reported [1].

Polymer molecularly imprinted matrices are often produced via polymerisation of a monomer in the presence of the template molecule. The monomer may be functionalised and interact with the template (e.g., via hydrogen bonds), be bound to it (via covalent or coordination bonds) or may be “inert” towards the template molecule. Similarly, many types of polymerisation processes have been employed for producing molecularly imprinted matrices, including both the classical chemical polymerisation reactions (e.g., radical, anionic, cationic) [2] and even electrochemical polymerisation [3]. Once the polymerisation process is complete, the template molecule is then removed from the matrix, leaving behind the template-shaped cavities.

Molecularly imprinted polymers (MIPs) have found wide application, particularly in sensing and biosensing, due to their high selectivity (translating into low cross-sensitivity to substances other than the intended analyte), high sensitivity to the intended analytes and fairly straightforward fabrication procedure, which does not involve as stringent purity requirements as in the case of inorganic semiconductor sensors [4]. The analytes, for which MIP-based sensors are reported, include a variety of small molecules, proteins, carbohydrates (particularly glucose) and even metal ions [5,6,7,8,9,10].

Among sensing applications, the use of molecularly imprinted conjugated polymers is of particular significance, as conjugated polymer matrices, due to their intrinsic conductivity, allow the fabrication of highly sensitive chemoresistive sensors. Although a variety of such sensors have been reported [11], polypyrrole appears to be the material, which has attracted the most research interest, likely due to its biocompatibility [12]. This is because it can be used for bio-sensing applications, unlike other conjugated polymers.

Polypyrrole (PPy) can be produced electrochemically via potentiostatic, amperostatic or potentiodynamic methods. In potentiostatic methods, the applied potential is typically in the range of +0.7–0.8 V versus a standard calomel electrode (SCE). Amperostatic methods, in turn typically carry the electrochemical polymerisation of pyrrole at a current density no higher than 10 mA/cm^2^ [13,14]. In the case of potentiodynamic methods, most commonly realised via cyclic voltammetry, the applied potential is scanned between −0.8 V and 1.4 V versus SCE [15,16].

During electrochemical polymerisation, regardless of the selected method, the anode is slowly covered with a layer of the incipient polypyrrole, which gradually precipitates out of the solution. The formed polymer undergoes oxidative doping during the process (as the oxidation potential of a conjugated polymer is always less positive than that of its respective monomer), resulting in an increase of its conductivity. The positive charge present on the oxidised polymer chains is balanced by the negative charge present on anions (originating from the electrolytes present in the solution), which are incorporated into the polymer layer forming at the electrode. The oxidised PPy is typically highly conducting, allowing relatively thick PPy layers to be produced via electrochemical polymerisation (due to the limited applied potential drop across the thickness of the film). Consequently, the polymer film on the electrode can act as an ion-exchanging matrix, with the anions present being “bound” to the matrix only electrostatically. Since those anions typically are small and highly mobile, they can be easily exchanged for other anions. It is also possible to produce a cation-exchanging polymer layer, as the doping level of the polymer determines not only its conductivity, but also affinity to anions and cations. It should be noted however, that most conjugated polymers can be irreversibly “over-oxidised”, e.g., through the application of excessively high potentials, resulting in damage to their chemical structure, i.e., partial degradation of the polymer film.

The key advantage of electrochemical polymerisation is the ability to precisely control the potential, at which the polymer film was deposited. Electrochemical polymerisation is quite commonly used when very thin layers of conjugated (co)polymers are desired, e.g., when manufacturing active layers for sensors. Electrochemical methods can also be perceived as a facile way of preparing conjugated polymer and copolymer films, when an investigation is aimed at establishing the “core” properties of a material, prior to attempting to tailor them for a particular application or when the scope of the investigation does not allow developing and optimising a synthetic procedure using only chemical agents. The amount of polymer produced via this method is however limited by the surface area of the employed electrode, therefore, larger amounts of PPy are typically produced via chemical polymerisation.

This work is intended as a tutorial review of the advances in sensors and biosensors based on electrochemically-produced molecularly imprinted polypyrrole (MIPPy). In the review, we sequentially introduce the issues and discuss the most relevant progress in designing MIP-based sensors, the methods for fabricating and investigating such sensors, the most relevant performance benchmarks and the applications, as well as the most relevant analytes targeted by MIP-based sensors.

## 2. Theoretical Considerations

In order to design a MIP-based sensor, theoretical methods, such as semi-empirical, ab initio and density functional theory calculations, are frequently used [17,18]. These methods are employed for tailoring the chemical and spatial structure, as well as the distribution of functional groups on the surface of the MIP so as to achieve the highest possible affinity between it and the template molecule, which is the intended analyte.

A good example of extensive work being done on the theoretical side of designing a molecularly imprinted polymer-based sensor is the development of L-tryptophan sensors based on PPy and polyaniline [18]. The first step was to use the Monte Carlo method to generate the possible structures of molecularly imprinted polymers, followed by the search for the conformers with the lowest energy. Next, the structures of the conformers were optimised and the electronic structures of those optimised conformers were predicted via the use of density functional theory. Once a set of low-energy conformers was chosen, potential energy surface scans of their structures were performed, to estimate the rotation probabilities between the aromatic rings of the conformers.

**Figure 1:**
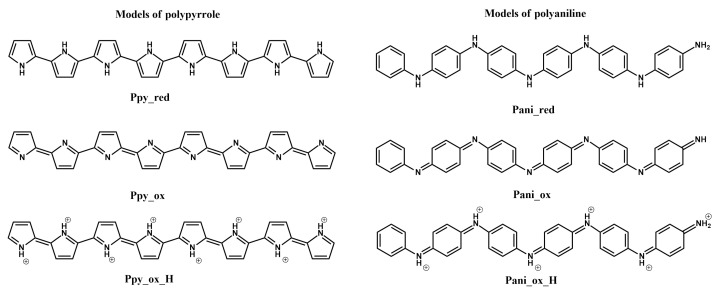
Structures of polypyrrole and polyaniline [18].

Based on these results, the various considered structures of PPy and polyaniline (Figure 1) were ranked in terms of their affinity for L-tryptophan, with PPy emerging as the most favourable structure in terms of its HOMO energy, based on which it was suggested that determination of tryptophan is only feasible with the use of PPy. Interestingly, electrochemically polymerised pyrrole was recommended to be peroxidised, in order to stabilise the imprinted polymer structure. Conversely, it was recommended for polyaniline-based molecularly imprinted polymers to be produced in a basic environment, which is noted as unlikely, due to the polymer being formed in strongly acidic environments and effectively making PPy the more favourable choice of polymer for molecularly imprinted L-tryptophan sensors [18].

Theoretical predictions, especially assisted by computers, can also be used to design composite materials for molecularly imprinted polymer sensors [19]. In a recent study, the interactions between the components of the composite, which included multi-walled carbon nanotubes, and the intended analyte, bisphenol A, particularly the conjugation of bisphenol A and the nanotube surface were predicted. To this effect, Hartree-Fock restricted at level 6-311G simulations were employed to predict the geometries of the bisphenol A and 3-aminopropyltriethoxysilane molecules and obtain their minimal energy conformation models. A pre-assembled bisphenol A—3-aminopropyltriethoxysilane system has been assumed manually and used as a basis for further simulations and geometry optimisations using Gaussian 09w software.

## 3. Sensor Fabrication Methods

**Figure 2:**
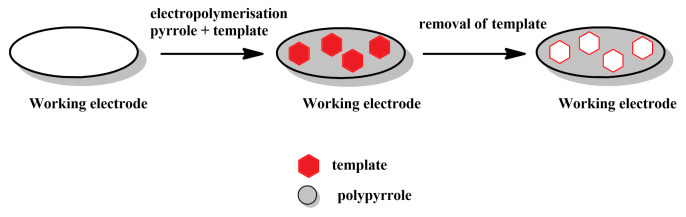
Schematic depiction of the stages comprising the simultaneous polymerisation and imprinting approach.

**Figure 3:**
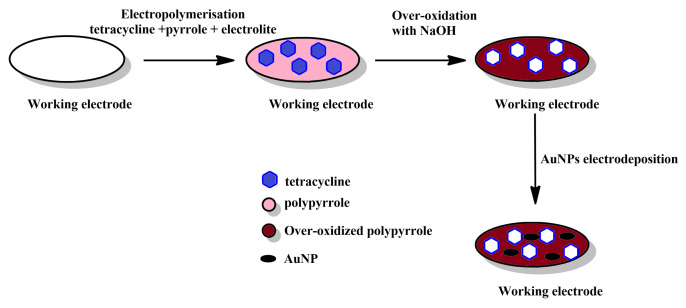
Schematic depiction of the stages comprising the simultaneous polymerisation and imprinting approach with over-oxidative post-treatment.

**Simultaneous polymerisation and imprinting (Figure 2):** In this, possibly most straightforward method for producing MIPPy layers, a solution that contains both pyrrole (or another, pyrrole-based monomer) and the desired template is treated electrochemically to induce electrochemical polymerisation and precipitation of the MIPPy layer on the working electrode. The template, can either interact with pyrrole or can be integrated into the PPy via occlusion. The precipitation is typically followed by the removal of the template, either via repeated rinsing with various chemicals (e.g., solvents, hydrolysis-inducing agents, etc.) or via simultaneous rinsing and electrochemical treatment. This may be followed by other post-treatment methods, aimed at restoring the activity of the sensor, as the over-oxidation typically results in lowering the conductivity of PPy (Figure 3).

PPy can be over-oxidised when highly positive potentials are applied to it. This causes it to lose conductivity to some extent, due to partial degradation of the conjugated bond system (e.g., introduction of conjugation breaks, ring opening and introduction of oxygen-bearing functionalities). Over-oxidation can also result in functional groups, such as carbonyl or carboxyl are incorporated into the main polymer chain, which can modify the affinity of PPy to positively- and negatively-charged species. In some cases this can result in increased selectivity, as has been found for dopamine. Simultaneously, the diminished conductivity of over-oxidised PPy can be alleviated to some extent by decorating the layer with golden nanoparticles, which can also act to increase the sensitivity of the sensor [20].

**Figure 4:**
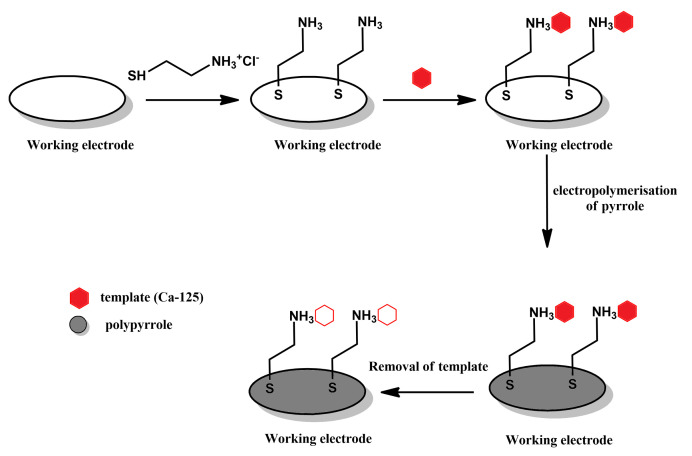
Schematic depiction of the stages comprising the polymerisation on template-modified electrodes approach.

**Polymerisation on template-modified electrodes (Figure 4):** Another approach to producing MIPPy layers is to perform electrochemical polymerisation in a solution of pyrrole on an electrode onto whose surface the template has already been deposited either via physical means or by chemical bonding (as illustrated in Figure 4 This is followed by removing the template from the MIPPy, typically by immersing the MIPPy-coated electrode in various liquids, such as an aqueous solution of HCl [21], in a solution of NaOH and H_2_O_2_ in a mixed acetonitrile/water solvent system [22] or in a mixture of acetonitrile and acetic acid [23].

In order to employ this method of producing MIPPy-based sensors, the surface of the electrode must first be modified, so as to allow the template to remain at this surface prior to the deposition of the PPy layer. In the case of developing a sensor for the CA-125 template (as presented in Figure 3), the working electrode was first modified with a self-assembled cystamine layer. The amine groups of cystamine were then used to covalently bind with the template molecule.

When developing a gp120 sensor, the Authors modified the electrode by depositing a graphene-like carbon nano fragment bismuth oxide composites, followed by dropwise coating of chitin onto the surface of the electrode and drying at room temperature. Such a modified electrode was activated by treating it with glutaraldehyde [24]. A similar approach was employed for the manufacture of α-synuclein sensors, where the electrode was treated with a solution of chitin and chitosan, followed by activating it with glutaraldehyde [25]. This method, as illustrated by the above examples, is primarily used for producing MIPPy-based sensors for detecting bio-active substances.

## 4. Detection of Contaminants

Sensors and bio-sensors based on MIPPy layers can be employed to detect the presence of a wide array of analytes, such as drugs, crop protection agents, byproducts of the plastic industry, heavy metal cations and others. These analytes can be detected in a variety of matrices, ranging from aqueous solutions, through consumer goods and even foodstuffs.

### 4.1. Detection of Antibiotics and Other Drugs

**Figure 5:**
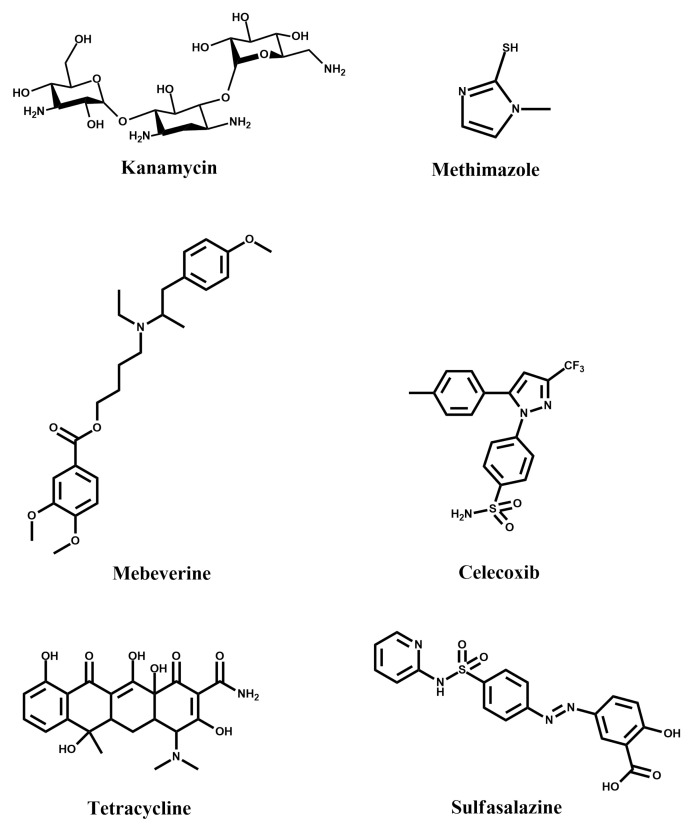
Structural formulae of some drugs that can be detected using MIPPy-based sensors.

Contamination by antibiotics and drugs is a concerning issue as it can promote the growth of drug-resistant bacteria [26,27]. Similarly, following the presence and concentration of a variety of drugs in medical samples is highly relevant for patient care, particularly when treatment involves the administration of multiple drugs. Consequently, MIPPy-based sensors, which are cost-efficient, sensitive (Table 1) and highly portable, are a great asset for monitoring the environmental contamination by these substances (Figure 5), as well as being excellent alternatives to many currently used methods in diagnostics, which involve sophisticated instruments and complex sample preparation protocols.

Kanamycin is an antibiotic for use in treating farm animals, but has several adverse effects on humans. Consequently, food safety regulations require its monitoring in various products, such as milk [28]. A MIPPy-based Kanamycin sensor was developed via electrochemical polymerisation of pyrrole from a solution also containing Kanamycin [21]. The MIPPy layer was deposited on a glassy carbon electrode, which was first coated in graphene oxide, in order to increase its effective surface area. Kanamycin was removed from the MIPPy-coated electrode via its immersion in 0.01 M hydrochloric acid.

Methimazole, similar to the abovementioned kanamycin, is a widely used drug. Its detection was achieved by a MIPPy-based sensor [29], deposited electrochemically on a graphite electrode. Differential pulse voltammetry was employed in order to ensure rmeoval of the template from the MIPPy layer. The sensor was found to be fairly selective, showing little current variation in the presence of comparable concentrations of interferents—drugs similar structurally to methimazole. A similar approach was taken for the detection of mebeverin, where a graphite electrode was used and where the template was also removed by voltammetric cycling [30]. This was also the case for celecoxib, in the case of which the only deviation from this procedure was that a different potential range was employed for the removal of the template via voltammetric cycling [31].

The use of electrochemical over-oxidation for the post-treatment of MIPPy-based tetracycline sensing layer is an interesting choice, particularly as a method for promoting the removal of the template molecule from the MIPPy matrix [32]. Although some loss of sensitivity was expected due to the treatment, Au nanoparticles were then deposited on the surface of the treated MIPPy player, in order to mitigate this issue.

Sulfasalazine is a drug for treating rheumatoid arthritis and other diseases. A MIPPy-based sulfasalazine bio-sensor has been developed for use in pharmaceutical samples. Interestingly, over-oxidative post-treatment was also used here, as a method for removing the template molecule from the PPy matrix [33].

### 4.2. Detection of Other Contaminants

**Figure 6:**
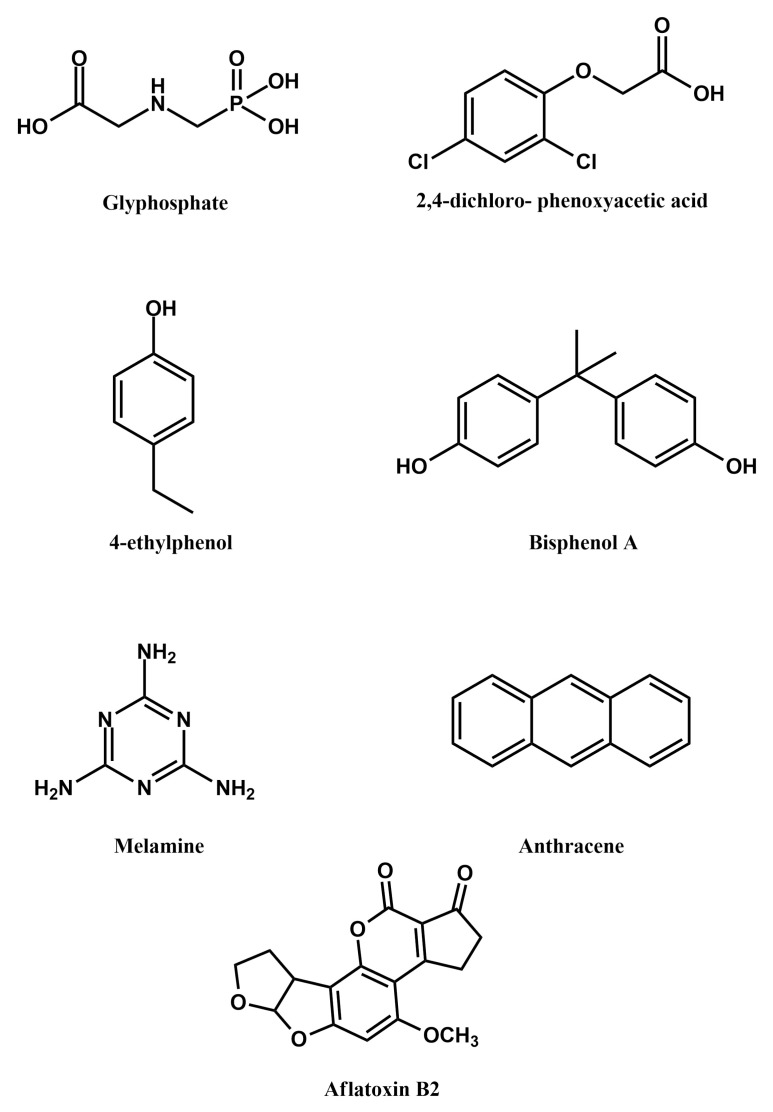
Structural formulae of some non-drug contaminants that can be detected using MIPPy-based sensors.

Among other contaminants, toxic analytes, regardless whether originating from the industry or from the natural environment, are the main area of focus for the application of MIPPy-based sensors (Figure 6). Consequently, the analysed matrices are most often water samples, as either groundwater contamination is suspected or, in the case of food-grade containers, water will be the primary agent responsible for leaching these contaminants out of the container material. Interestingly, likely due to the fact that the analyte molecules are on average smaller than in the case of drugs, significantly higher sensitivity is reported for such analytes (Table 2) than for drugs.

Glyphosate is a broad-spectrum herbicide that is quite widely used in agriculture, due to its relatively low toxicity and high efficacy. When used excessively, however, it can accumulate in soil and groundwater, as well as in some crops, causing adverse effects to humans. A sensor for determining glyphosate levels in water and in cucumbers has been reported, based on MIPPy. The sensor was fabricated using a bare gold electrode, onto which a MIPPy layer was deposited by electropolymerisation of pyrrole in the presence of glyphosate. The template was removed by over-oxidising PPy in 0.1M sodium hydroxide [34].

Similarly, 2,4-dichlorophenoxyacetic acid is often used as a systemic herbicide, which can contaminate groundwater. An alarming observation is that its concentration in groundwater has been found to be gradually increasing over recent years [35], which is a potential human health issue. Consequently, reports of its sensitive, on-site detection methods, such as the recently developed MIPPy-based sensor [36], are of significant interest for monitoring the presence of this compound in groundwater. A pencil graphite electrode, which was rinsed with ethanol and water, was used for the polymerisation of pyrrole in the presence of 2,4-dichlorophenoxyacetic acid. The template was removed from the MIPPy layer by treating it with sodium hydroxide, as in the case of glyphosate mentioned above.

The presence of 4-ethylphenol in wine is undesirable, as it is a compound, which is produced when wine is contaminated with Brettanomyces/Dekkerabruxellensis yeast, during their enzymatic reduction of p-coumaric acid. The compound adversely affects the quality of wine, due to its unpleasant taste. A MIPPy layer was deposited on a glassy carbon electrode, followed by treating the layer with an ethanolic solution of sodium hydroxide to remove the template from the MIPPy matrix [37].

Bisphenol A is a toxic chemical widely used in the plastics industry, due to which its concentrations need to be monitored, both in the industry and in the environment. A electrochemically-fabricated MIPPy-based sensor has, therefore, been developed for detecting bisphenol A. The sensor was produced by depositing the MIPPy layer on a laser-scribed graphene electrode, followed by removal of the template via immersing the sensor in a mixture of acetic acid and methanol [7].

Similar to bisphenol A, melamine is another pollutant originating mainly from the plastics industry. Due ot the toxicity of melamine, it is important to monitor the contents of food-grade containers for possible contamination with melamine, released from the container material. Currently used methods for detecting melamine are complex or require significant instrumentation (e.g., detection via liquid chromatography, IR spectroscopy, etc.), while involving a lengthy sample preparation procedure. Consequently, the reported MIPPy-based melamine sensor, which may allow detection to be achieved directly, in a melamine-containing matrix, is particularly interesting. In this case, the MIPPy layer was deposited onto a glassy carbon electrode modified with graphene oxide, with the template removal being realised by immersion in a solution of hydrogen peroxide and sodium hydroxide in a mixed water-acetonitrile solvent [22].

An interesting application of molecularly imprinted polymers is for the detection of heavy metal ions, such as mercury (II) cations [38]. Sensors were fabricated on Au transducers, decorated with ZnO nanorods, using both imprinted and non-imprinted polypyrrole, with L-cysteine being present in the polymerisation solution and being incorporated into the PPy, in order to act as a mercury chelating agent. The presence of cadmium (II) cations in water can also be detected via this method [39]. The reported sensor utilises a glassy carbon electrode, whose surface has been modified with reduced graphene oxide and onto which polypyrrole has been electrochemically deposited from a solution containing the template.

Anthracene is one of the compounds belonging to the group of policyclic aromatic hydrocarbons, which are suspected of being carcinogenic. Of those compounds, anthracene is commonly found as a pollutant in both the atmosphere and aqueous media. Contemporary methods of detecting anthracene in such environments rely on gas chromatography and mass spectrometry, however, these methods require cost-intensive instruments that have low portability, limiting the possibility of performing on-site measurements. The reported MIPPy-based anthracene sensor can serve to avoid the aforementioned issues, providing a portable sensing solution. The template was removed from the MIPPy-coated glassy carbon electrode via voltammetric cycling [40].

Aflatoxins are a highly toxic class of fungal secondary metabolites, mostly produced by Aspergillus flavus and Aspergillus parasiticus. Aflatoxin B2 is one of those compounds and has been used as a template for molecular impinting [41]. A more elaborate approach was used in this case, as one type of sensors involved a composite, which was composed of chitosan- and aflatoxin B2-coated ZnO nanoparticles were deposited on gold electrodes. These electrodes were then coated with polypyrrole via electropolymerisation and the template was removed by extraction with a mixture of methanol and acetic acid. For the other type of sensors, pyrrole was electrochemically polymerised on gold electrodes in the presence of ZnO nanoparticles coated with aflatoxin B2 and chitosan. The aflatoxin B2 was removed from the MIPPy via electrochemical over-oxidative treatment of the modified electrode in a Na_2_HPO_4_ solution.

## 5. Detection of Bio-Active Substances

**Figure 7:**
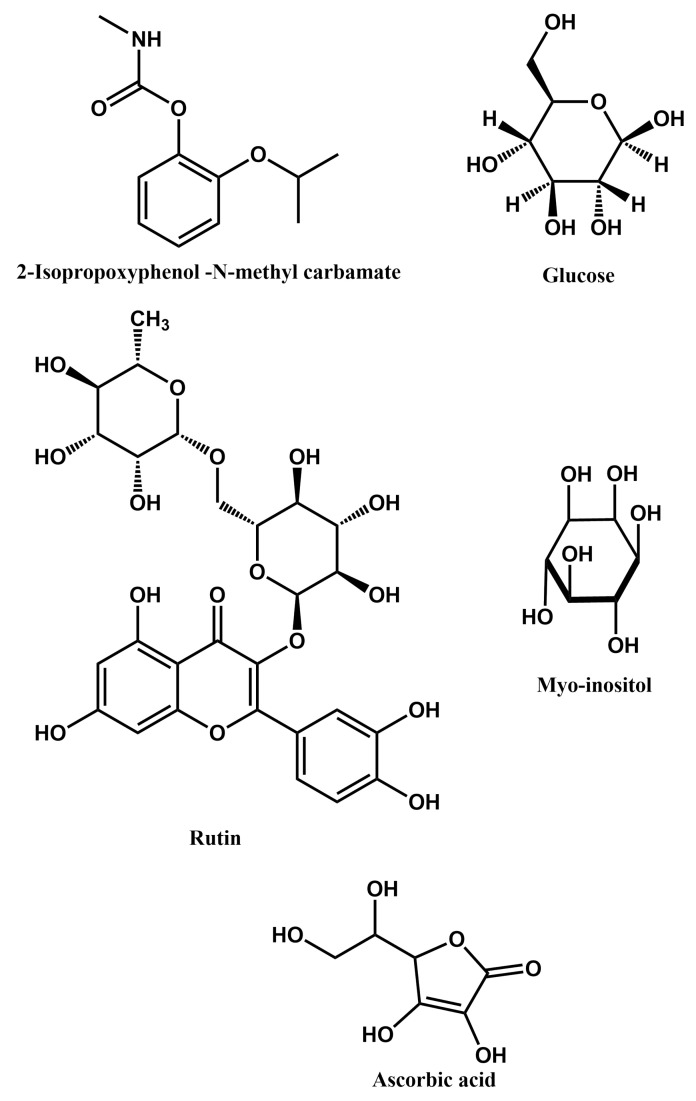
Structural formulae of some bio-active substances that can be detected using MIPPy-based sensors.

Clinical diagnostics, as well as screening for exposure to various toxins, are heavily reliant on monitoring a variety of bio-active substances, mostly metabolites (Figure 7). Consequently, the vast majority of analytes are investigated in the various human bodily fluids. Due to the complexity of such matrices, the samples require elaborate preparation, handling, storage and treatment protocols, often involving non-standard conditions, such as cryogenic storage. Furthermore, these procedures are often specific to each analyte, necessitating the harvesting of numerous samples to perform the desired diagnostics. The use of MIPPy-based sensors can alleviate this issue, as apart from interacting with their respective “template”, the sensors do not significantly affect matrices, making it feasible to reuse such samples. Not only does this allow harvesting smaller samples, which is less troubling for the patient, but also does not result in any significant loss of sensitivity as compared with the traditional methods (Table 3).

2-Isopropoxyphenyl-N-methyl carbamate is a widely used insecticide, which has been classified as moderately hazardous by the World Health Organisation. The major metabolite of this chemical compound in mammals is 2-isopropoxyphenol. Consequently, the presence of 2-isopropoxyphenol in biological samples can be used as a bio-marker to exposure to the toxic insecticide. A 2-isopropoxyphenol sensor is reported, based on a MIPPY layer deposited on a bare glassy carbon electrode. In order to remove the template from the PPy matrix, the electrode was immersed in a 1:5 (*v*/*v*) mixture of acetic acid and acetonitrile. The optimal electrode immersion time was found to be approx. 10 min, as by this time all the adsorbed template was removed from the MIPPy layer [23]. This sensor can operate in both a differential pulse voltammetric regime and in a cyclic voltammetry regime.

Detection of glucose has also been reported to be realised via the use of MIPPy-based bio-sensors [42]. Such a solution is interesting and appears to be favourable in comparison with the non-reusable strips used in glucometers, with the results of such measurements being significantly affected by changes in the temperature, and humidity of the environment, as well as the pH and ionic strength of the blood matrix. The sensor was produced using a platinum screen-printed electrode, via a standard electropolymerisation procedure. The template was then removed by immersing the electrode in a 5% acetic acid. Glucose concentrations were tested by depositing serum samples dropwise onto the sensor, with its response to increasing glucose concentrations being linear in a range of 0.02–8 mg/mL.

Rutin is a glycoside found in many plants and particularly in their leaves. It has an array of beneficial effects on human health, such as being an anti-inflammatory, anti-bacterial, anti-tumor and anti-oxidising agent. In medical applications, rutin can be employed to regulate capillary permeability and stabilize blood platelets. Consequently, its detection is relevant for the purpose of monitoring human health and the reported MIPPy-based rutin sensor can find wide application, possibly even for lab-on-a-chip type solutions. A glassy carbon electrode was used for preparing this sensor. It was first modified by depositing a reduced graphene oxide—carbon dots composite onto its surface and then annealed at 50 ${}^{\circ }$C. This modified electrode was used for polymerisation of pyrrole in the presence of rutin, followed by removal of this template by immersing the electrode in a sodium hydroxide solution [43].

Glycoprotein gp120 is an important biomarker for diagnosing the HIV-1 virus. It is believed that its binding with the CP4 receptor on the surface of human cells may be a key factor for developing a therapy directed against HIV-1 [44]. A gp120 bio-sensor was developed by covalently binding gp120 to the surface of a glassy carbon electrode, followed by activating it using glutaraldehyde and coating the electrode with PPy via electrochemical polymerisation [24]. Although the MIPPy layer was then treated with methanol and acetic acid, in order to remove gp120 from it, the efficacy of this removal has not been investigated and remains questionable, particularly in light of the large gp120 molecules being covered by a PPy layer, making the sensing mechanism itself debatable. The surface of the sensors was studied by scanning and transmission electron microscopy, revealing the presence of cracks on the surface of the sensors. Another sensor has also been developed, utilising a small-molecule entry inhibitor, NBD-556, in conjunction with the other components of the previous sensor. The addition of NBD-556 resulted in an increased sensitivity towards detecting gp120.

Early diagnostics of subclinical mastitis in dairy cows rely on monitoring serum amyloid A levels in milk. While such mastitis does not manifest visible external symptoms, it significantly reduces the milk production of afflicted cow herds and promotes the development of other diseases. Consequently, a serum amyloid A sensor has been developed, utilising a MIPPy layer, with the imprinting being conducted by first depositing the amyloid A onto the electrode, followed by coating it in polypyrrole. In order to remove the template, the electrode was immersed for an hour in an acetic acid solution [19].

Prostate-specific antigen is also a biomarker for cancer diagnostics (prostate cancer). The detection of this biomarker, however, is fairly problematic and primarily relies on immunoassays, e.g., enzyme-linked immune-absorbent assay, which are fairly cost-intensive and involve complex sample handling and preparation procedures. Consequently, the recently reported prostate-specific antigen sensor [45] appears to be a more elegant, straightforward approach. The sensor was fabriacted on a gold screen-printed electrode, which was first treated by subjecting it to voltammetric cycling in a sulfuric acid solution. Following polymerisation, the template was removed by treating the electrode in oxalic acid.

Pyrrole has also been electrochemically polymerised in the presence of a carbohydrate antigen (CA-125), which is an important serum biomarker for ovarian cancer detection). In this work, the MIPPy layer was deposited on a gold electrode through a four-stage process [46]. Interestingly, in this case, the CA-125 layer was first deposited on the electrode (via an adsorbed cysteamine layer) and then coated with a layer of electrochemically produced polypyrrole. Although the Authors have attempted to remove the CA-125 by immersing the sensor in sodium dodecyl sulfate and deionised water, the efficacy of its removal is yet to be confirmed.

Myo-inositol a polyalcohol that plays an important role in monitoring the treatment of various diseases, such as diabetes [47]. A MIPPy-based sensor was produced [48] using a glassy carbon electrode that has been coated with nickel-doped reduced graphene oxide. The electrode was immersed in a sodium hydroxide solution to remove the template and the MIPPy layer was then treated electrochemically, to induce over-oxidation of PPy.

α-Synuclein is a 140-amino acid (14.3 kDa) protein that expressed in presynaptic and perinuclear regions of the central nervous system. Previous studies have proven that it has a close link with the pathogenesis of Parkinson’s disease [49]. Consequently, bio-monitoring of this protein can be a step forward in early diagnostics of Parkinson’s. For this purpose, a MIPPy bio-sensor for sensing α-synuclein in blood has been developed [25]. The MIPPy layer was deposited on a glasy carbon electrode, which was modified with graphene nanosheets and treated with a conjugated guanidinium-based polymer, with chitosan and with glutaraldehyde. After its electrochemical deposition, the MIPPy layer was treated with sodium hydroxide to extract the template, although the efficacy of removal was not verified.

Ascorbic acid (Vitamin C) is an important bioactive substance, involved in many metabolic pathways. Recently, a MIPPy-based ascorbic acid sensor was reported [50], utilising an uncommon deposition of the MIPPy layer onto a glassy carbon electrode coated with PEDOT nanorods, which in turn were decorated with black phosphorous quantum dots. The sensor was used to detect Vitamin C in various soft drinks, showing good performance.

Neuron-specific enolase is one of the key enzymes in the glycolytic pathway and its elevated levels are often used as a symptom of various health issues, such as neuronal damage and small cell lung cancer. This enzyme is typically monitored via radioimmunoassay, enzyme-linked immunosorbent assay and fluoroimmunoassay, all of which are either lengthy or involve costly instruments and reagents. Consequently, the reported MIPPy-based neuron-specific enolase sensor is quite promising for the development of diagnostics [51]. Interestingly, the sensor utilised a gold nanoelectrode array deposited onto a glassy carbon electrode and treated with sulfhydryl-functionalised monomers, supposed to act as anchoring sites for the MIPPy layer. The layer itself was deposited via electropolymerisation from a solution containing a pyrrole-functionalised ionic liquid and neuron-specific enolase. The resultant sensor was treated with acetic acid and sodium dodecyl sulfate, in order to remove the template.

## 6. Conclusions

Conjugated polymers are frequently used receptor layer materials for various types of sensors. In many applications, however, the selectivity of these materials is rather lacking—this is particularly the case for chemoresistive sensors that rely on the analyte affecting the conductivity of the polymer by its doping/dedoping. In contrast, the use of molecular or ionic imprinting methods greatly alleviates this drawback, allowing highly selective and even specific sensors to be developed.

Among the reviewed works, great focus is attached to analytes in biological systems, both metabolites and bio-active substances. This ties in both with the biocompatibility of polypyrrole and therefore the possibility of developing implantable bio-sensors and with the ever-current need for fast and reliable diagnostics and screening.

Despite the above, other types of analytes are also represented, including crop protection agents and even heavy metal ions. This goes to show that the MIPPy-based sensor combine the best features of conjugated polymers—their versatility, facile layer manufacture and ability to react to various species—and molecular imprinting methodology—high selectivity and great sensitivity. As such, this type of sensors has great potential for future applications, both in biomedical and environmental monitoring.

The development of electrochemical bio-sensors utilising molecularly imprinted polypyrrole layers as the receptor layers is gaining increasing research attention. This is due to the advantages offered by MIPPys: the ability to adapt the production method to a particular application, their excellent chemical and thermal stability, low cost of polymers and relatively high energy-efficiency of producing such sensors. There are several examples of sensors based on MIPPy layers that have been developed recently and exhibit LOD values and linear operating ranges sufficient for application as bio-sensors. The majority of sensors, however, involves sophisticated fabrication methods. The primary issue in fabricating MIPPys is the insufficient stability of natural template molecules in the conditions, in which the MIPPy layers are produced (i.e., solvents for the polymer and removal of the template).

In our opinion, MIPPy-based sensors have great potential as solutions for current (bio-)sensing needs, such as clinical point-of-care testing or at home monitoring and self-diagnostics kits, is the need to optimise the methods for producing such sensors. However, overcoming the drawbacks of electrochemical polymerisation (i.e., difficulty in up-scaling and achieving high productivity in terms of mass of the MIPPy produced from an electrolysis unit in a unit of time), polymerisation in bulk (heterogeneous bonding sites) and many other synthetic methods (poor synergy with electrochemical detection techniques) are the key challenges to be resolved. Practically, it is a challenge to develop methods that will be able to yield sensor platforms, which will achieve biologically-relevant detection properties, and simultaneously allow mass producing such sensors. Taking into consideration the advantages offered by MIPPys to sensing platforms, we expect that this area of research will, in the future, only see an increase in the research interest dedicated to developing it further.

## Figures and Tables

**Table 1:** Drug sensing performance of MIPPy-based sensors.

Template	Operating Range	LOD ^1^	Matrix	Interferents	Methods	Ref
Kanamycin	5 nM–1 μM	5 nM	Milk sample, kanamycin standard sample	-	impedimetric analysis	[21]
Methimazole	7 μM–6 mM	3 μM	Water, Human blood serum	Thiacetamide, Thiourea, Benzimidazole, Diazepam, 4-dimetylaminopyridine, ticlopidine hydrochloride	DPV	[29]
Mebeverine	10 nm–1 μM, 10 μM–1 mM	8.6 nM	spiked human blood serum	asdonpezile, chloroquine, levertiracetan, phexophenadine, naproxen, mequinol	DPV	[30]
Celecoxib	5 nM–20 μM	2.34 nM	spiked human blood serum	fluoxetine, pantoprazole, tetracycline, acetaminophen, famotidine, letrozole	CV, DPV	[31]
Tetracycline	1–20 μM	0.65 μM	spiked in shrimp sample	amoxicillin, chloramphenicol, oxytetracycline	DPV	[32]
Sulfasalazine	1.0–10 ppm	0.265 ppm	pharmaceutical sample	ascorbic acid, sulfadiazine, sulfamethoxazole	DPV	[33]

^1^ LOD—limit of detection.

**Table 2:** Performance of MIPPy-based sensors for detecting of non-medical pollutants.

Template	Operating Range	LOD	Matrix	Interferents	Methods	Ref
Glyphosate	5–800 ng/mL	0.27 ng/mL	spiked cucumber samples, spiked tap water	chlorpyrifos, aldicarb, aminomethyl phosphonic acid	DPV	[34]
2,4-Dichloro- phenoxyacetic acid	0.06–1.25 μg/L	0.02 μg/L	spiked drinking water	atrazine	EIS	[36]
4-Ethylphenol	0.2–34.8 μM	0.2 μM	wine samples	4-ethylguaiacol, dopamine	DPV	[37]
Bisphenol A	0.05–20 μM	8 nm	tap water. bottle water, drinking bottle, polycarbonate	estradiol, epinephrine, dibutyphtalate, gallic acid, caffeic acid, 4-chlorophenol, bisphenol F	[7]
Melamine	4.0–240 nM	0.83 nM	spiked milk	glycine, phenylalanine, tryptophan, histidine, tyrosine, ascorbic acid	EIS	[22]
Hg^2+^	-	1 pM	water/KCl	lead, cadmium and copper ions	Square wave woltammetry	[38]
Cd^2+^	1–100 μg/L	0.26 μg/L	water samples	Mn^2+^, Cr^6+^, Mg^2+^, Zn^2+^, Cr^3+^	Square wave anodic stripping woltammetry	[39]
Anthracene	-	12 nM	mineral water	sodium, potassium and calcium cations, nitrate, sulfate, chloride and carbonate anions	square wave voltammetry	[40]
Aflatoxin B2	-	0.2 fg/mL	milk	-	EIS, DPV	[41]

**Table 3:** Bio-active substance sensing performance of MIPPy-based sensors.

Template	Operating Range	LOD	Matrix	Interferents	Method ^1^	Ref
2-Isopropoxyphenol	0.21–75 μM	0.21 μM	biological fluids	chlorferon, disulfoton-sulfone, fenamiphos, strychnine	DPV	[23]
Glucose	20–800 mg/dL	6.064 mg/dL	serum sample	-	CI ^2^	[42]
Rutin	0.01–6.5 μM	3 nM	human serum	apigenin, primuletin, luteolin	DPV	[43]
Glycoprotein gp120	0.1–50 ng/mL	0.015 ng/mL	spiked human serum	HIV-p24, hCH, CEA	DPV	[24]
Amyloid A	0.01–200 ng/mL	5 pg/mL	spiked milk	α-lactalbumin, β-lactoglobulin, casein, bovine serum albumin	DPV	[19]
Prostate-specific antigen	0.01–4 ng/mL	2 pg/mL	human serum	-	DPV	[45]
Carbohydrate antigen (CA-125)	0.01–500 U/mL	0.01 U/mL	serum	compound found in artificial serum	square wave voltammetry	[46]
Myo-inositol	0.1–10 nM	76 pM	sugarcane vinasse	L-arbitol, D-mannitol, erythritol, glucose	DPV	[48]
α-Synuclein	0.1 pg/mL–8 ng/mL	0.035 pg/mL	blood	-	DPV	[25]
Ascorbic acid	0.01–4 mM	3.3 μM	soft drinks	-	DPV	[50]
Neuron-specific enolase	0.01–1 ng/mL	2.6 pg/mL	Clinical serum samples	-	DPV	[51]

^1^ Method used for ascertaining the response of the sensor to the analyte. ^2^ Chronoimpedimetric.

## Data Availability

Not applicable.
